# Supplementary material for: Overexpression of LjPLT3 Enhances Salt Tolerance in Lotus japonicus
Source: Int J Mol Sci. 2023 Mar 8;24(6):5149. doi: 10.3390/ijms24065149 (PMC10048936; doi:10.3390/ijms24065149)
Supplement: Supplementary file 1 [file ijms-24-05149-s001.zip › ijms-2228826-supplementary.pdf]

Table S1 Primers used in this study.

Genes	Forward primer (5'-3')	Reverse primer (5'-3')
**Primers used for gene cloning**		
LjPLT3-OE	GGTACCTGTCTACTCAAGAAAAAAACAACCC	GGATCCAGGCTTCACTAGTTTGCACCTCA
pLjPLT3:GUS	AACTGCAGCAACAATAAATTCATCACTAG	CGGAATTCTATCCTGCTTGTATGATTGAA
**Primers used for quantitative real-time analysis (qRT-PCR)**		
LjPLT3	CGTCACTATTATATCTGCCATATTCGG	CACTTTTCTCGCCTCGCCTACG
LjCAT	GCGCCTGACAGGCAAGATA	TGCCCAAGAGAACGATCAGC
LjSOD	TGAACAATGGTGAAGGCTGTG	CCTTGACATTATCGCTGCTGC
LjAPX	CCAACCCTCACATCTTCGACA	ACCTTCCTTCTCACCGGTCAA
LjGR	CGTGGTTGTGTTCCCAAAAAGA	GCATCCTGAAGTTCACCACCAT
LjACTIN	TGTGCGGAGGTACTTGGGCTTAAGA	CTTCCCAGGCAAATGCCAACACTAG

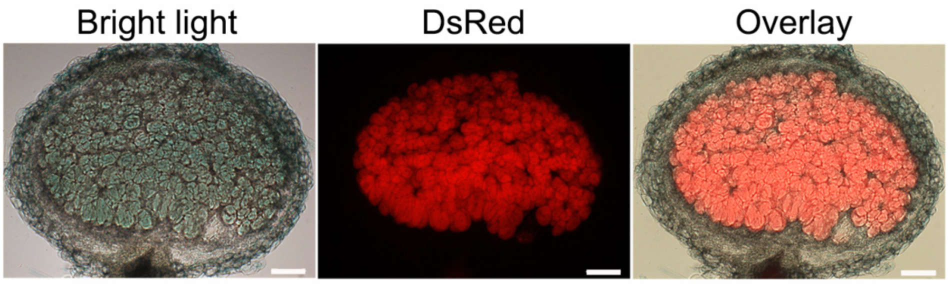

Figure S1. GUS staining of *pLjPLT3: GUS* plant. Cross-section of a nodule from a pLjPLT3: GUS plant showing GUS activity overlapped with bacteriods carrying *DsRED* in the infection zone. Three-day-old plants were inoculated with *M. loti* expressing *DsRed* and six nodules were collected for section four weeks later after infection. Scale bar=50 μm

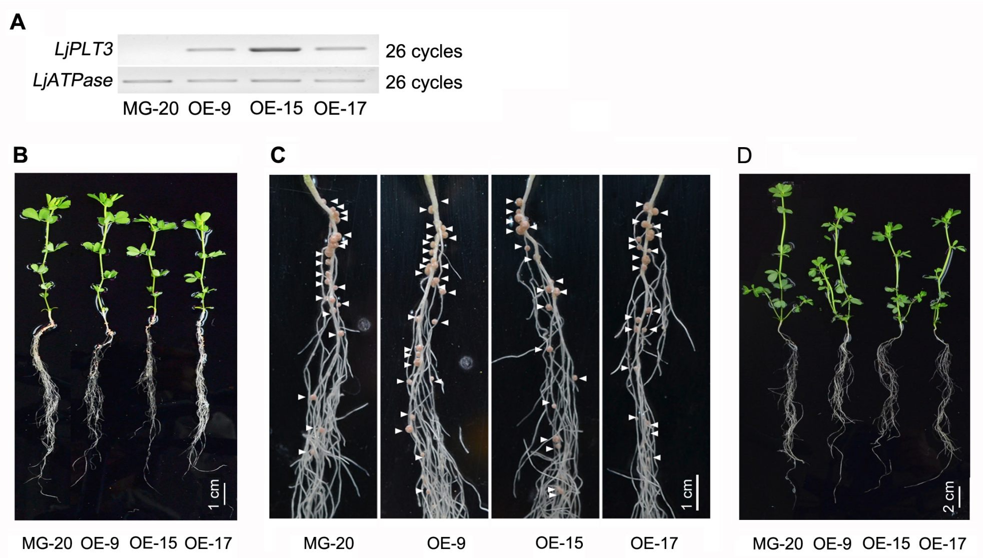

Figure S2. Phenotype of *OEPLT3* plants. (A) The expression level of *LjPLT3* gene in transgenic plants. (B) Four-week-old plants in symbiotic condition. (C) Nodules on the roots of transgenic plants and the wildtype. Three-old-day seedlings were inoculated with *M. loti* and representative plants were chosen for photograph four weeks later in (B) and (C). (D) Four-week-old plants in nitrogen-sufficient condition. 3-day-old seedlings were irrigated with Hoagland's solution once a week and representative plants were chosen for photograph four weeks later.
